# Pollen Aquaporins: The Solute Factor

**DOI:** 10.3389/fpls.2016.01659

**Published:** 2016-11-09

**Authors:** Juliana A. Pérez Di Giorgio, Gabriela C. Soto, Jorge P. Muschietti, Gabriela Amodeo

**Affiliations:** ^1^Instituto de Investigaciones en Ingeniería Genética y Biología Molecular – Consejo Nacional de Investigaciones Científicas y TécnicasBuenos Aires, Argentina; ^2^Instituto de Genética Ewald A. Favret – Centro de Investigación en Ciencias Veterinarias y Agronómicas – Instituto Nacional de Tecnología Agropecuaria – Consejo Nacional de Investigaciones Científicas y TécnicasBuenos Aires, Argentina; ^3^Departamento de Biodiversidad y Biología Experimental, Facultad de Ciencias Exactas y Naturales, Universidad de Buenos AiresBuenos Aires, Argentina; ^4^Instituto de Biodiversidad y Biología Experimental y Aplicada – Universidad de Buenos Aires–Consejo Nacional de Investigaciones Científicas y TécnicasBuenos Aires, Argentina

**Keywords:** aquaporin, fertilization, membrane intrinsic protein, solute permeability, plant fitness, pollen germination, water channel, water and solute transport

## Abstract

In the recent years, the biophysical properties and presumed physiological role of aquaporins (AQPs) have been expanded to specialized cells where water and solute exchange are crucial traits. Complex but unique processes such as stomatal movement or pollen hydration and germination have been addressed not only by identifying the specific AQP involved but also by studying how these proteins integrate and coordinate cellular activities and functions. In this review, we referred specifically to pollen-specific AQPs and analyzed what has been assumed in terms of transport properties and what has been found in terms of their physiological role. Unlike that in many other cells, the AQP machinery in mature pollen lacks plasma membrane intrinsic proteins, which are extensively studied for their high water capacity exchange. Instead, a variety of TIPs and NIPs are expressed in pollen. These findings have altered the initial understanding of AQPs and water exchange to consider specific and diverse solutes that might be critical to sustaining pollen’s success. The spatial and temporal distribution of the pollen AQPs also reflects a regulatory mechanism that allowing a properly adjusting water and solute exchange.

## Introduction

Over the last 25 years overwhelming evidence has been gathered indicating that the role of certain members of a complex superfamily of major intrinsic proteins (MIPs) known as aquaporins (AQPs) is to facilitate the permeation of water and small uncharged solutes (including gasses) through biological membranes (Bienert and Chaumont, 2014; Kitchen et al., 2015; Maurel et al., 2015; Pommerrenig et al., 2015). The first water channel -CHIP28, later named AQP1- confirmed not only the appearance of an unequivocally facilitated water path in a cell membrane (Preston et al., 1992) but also the role of the membrane osmotic permeability (*P*_f_), *i.e.*, that the membrane expressing these proteins can increase their capacity to exchange water 10- to 100-fold, reflected in a change in this parameter. The “simple permeability hypothesis” holds that AQPs act as key modulators of the *P*_f_ of membranes and sustains that the change in the rate of water transport is the critical trait for certain biological processes (Hill et al., 2004; Hill and Shachar-Hill, 2015).

Unlike ion channels, AQPs are small (∼30 kDa) tetramers in which each monomer is a functional unit. Although assembled primarily as homotetramers, certain groups in both animals and plants can form heterotetramers (Verbavatz et al., 1993; Neely et al., 1999; Fetter et al., 2004; Yaneff et al., 2014). The integral membrane region of each monomer is composed of six transmembrane α-helices, three extracellular loops (A, C, E) and two intracellular ones (B, D). The channel “signature” is two opposing and highly conserved NPA motifs (Asn-Pro-Ala) near the center of the molecule resulting from the dipping of two inverted hemi-helices on loops B and E (Murata et al., 2000; Ho et al., 2009; Tani et al., 2009; Wree et al., 2011). While this constriction zone is associated with the single-file conductance of water, a second constriction -typically composed of aromatic residues and an arginine known as the ar/R zone, is located in the outer channel vestibule and forms a strong selectivity filter that determines the solutes that can permeate through the pore (Fu et al., 2000; Sui et al., 2001; Beitz et al., 2006; Fu and Lu, 2007; Almasalmeh et al., 2014). Recent structural studies of AQPs from a diverse range of organisms have revealed new insights into selectivity and modes of regulation, including gating and trafficking (Kreida and Törnroth-Horsefield, 2015).

Genome and transcriptome sequencing data available from all kingdoms have confirmed a vast number of orthologous channels, challenging the classical outlook to address water/solute transport through biological membranes (Gupta et al., 2011; Alleva et al., 2012; Soto et al., 2012; Pérez Di Giorgio et al., 2014). MIP genes are particularly prevalent in the plant kingdom with 35–60 MIP isoforms in vascular plants compared to 10 in mammals including humans. Moreover, compared to other kingdoms, plants show not only a strong diversification (seven major classes have been distinguished: PIP, HIP, XIP, TIP, NIP, GIP, and SIP; this number is reduced to five or four in higher plants) but also higher gene copy numbers within certain species. Because “orthodox” AQPs were first suggested to facilitate the passive transport of water across cell membranes in response to osmotic gradients, paradigms to explore plant water hydraulics were opened. New research shed light on their impact on plant transpiration (Maurel et al., 2016; Yaaran and Moshelion, 2016), on solute transport including toxic metalloids (Bienert and Jahn, 2010; Pommerrenig et al., 2015) and gas transport (Herrera and Garvin, 2011; Bienert and Chaumont, 2014; Kaldenhoff et al., 2014).

In this context, the study of plant AQPs has been expanded to include specialized and symplastically isolated cells such as guard cells or pollen tubes in which the rate of water and solute exchange is critical to accomplishing their physiological task (volume change strategy: to swell or to grow). In the particular case of pollen tubes, growth is fast and complex, reflected by a spatial and temporal regulation tightly linked to the cellular process. Thus, pollen water status can be addressed as a structural, physiological and molecularly coordinated mechanism. It has been proposed that the identification of potential “water homeostasis control points” might improve our understanding of pollen quality and function upon exposure to environmental stresses (Firon et al., 2012).

Under this scenario, AQPs were considered natural candidates for controlling and fine tuning these control points. Analysis of *Arabidopsis* gene expression has helped to identify the genes responsible for pollen hydration and growth. Interestingly, pollen exhibits a fewer number and more exclusive types of AQP-expressed genes when compared to other single cell transcriptional profiles (Soto et al., 2008). Unlike many other cells, the AQP machinery in *Arabidopsis* mature pollen lacks plasma membrane intrinsic proteins and is restricted to a limited variety of members of other MIP subfamilies: tonoplast intrinsic proteins (TIPs) and NOD26-like intrinsic proteins (NIPs) (Honys and Twell, 2004; Pina et al., 2005; Wang et al., 2008; Qin et al., 2009; Loraine et al., 2013).

The aim of this review is focused on integrating information provided by available data in the field of pollen AQPs not only to highlight their physiological role but also to contribute to the understanding of their intrinsic properties.

## Pollen Hydration and Germination

Pollen grains undergo a sophisticated developmental program that includes internal cell adjustments during the different phases of dehydration and rehydration. These processes allow it not only to achieve fertilization as a final goal but also to cope with hostile environmental conditions. When a compatible pollen grain contacts the suitable stigma surface, it rapidly germinates and turns into an elongating pollen tube that will search for the ovules. Signaling molecules and ion channels act as pacemakers of the growth rate as well as controllers of the direction of the pollen tubes (Guan et al., 2013). Changes during germination and pollen tube growth result in mechanical stress sustained by the coordinated activity of the protoplasm and barriers (membranes and cell wall). Water uptake is thus critical during pollination: *i.e.*, rehydration on the stigma surface and pollen tube growth, not only because they demand water entry but also because pollen tube growth is a very fast process within plant cells. Volume increase is a consequence of water and nutrient uptake. The process is osmoregulated and pollen tube growth is so tightly synchronized to the osmolality of the medium (Benkert et al., 1997) that it can be completely arrested if osmolality is changed (Pierson et al., 1994). Inappropriate pollen hydration inhibits fertilization triggered by premature germination within the anther (Johnson and McCormick, 2001) or landing on an incorrect surface (Lolle et al., 1998). Despite the evidence for water uptake and osmoregulation during this process, data on water transport or on the specific role of AQP-mediated water transport were acknowledged much later than ion and solute transport (Sommer et al., 2008).

## Pollen Aquaporins: Only Four AQP Genes are Pollen-Specific

The first reports in the literature describing putative pollen water channels were performed in *Brassica, Nicotiana*, and *Lilium* (Marin-Olivier et al., 2000; Dixit et al., 2001; Bots et al., 2005a,b; Sommer et al., 2008). Interestingly, these first reports did not conclusively demonstrate that PIPs, the better-described “orthodox” water channels, were highly represented. Two PIPs were found differentially expressed in *Nicotiana* anther and stigma (Bots et al., 2005a,b). In *Brassica* pollen, the presence of PIPs was not clear (Marin-Olivier et al., 2000; Dixit et al., 2001). Comparative analysis of pistil transcriptomes revealed the expression of *PIP1* and *PIP2* in species with dry and semi-dry stigmas (*Oryza sativa, Crocus sativus, Arabidopsis thaliana*, and *S. squalidus*), but not with wet stigmas (*Nicotiana tabacum*) (Allen et al., 2010). Recently, stigmatic papilla cells transcriptome analysis in *A. thaliana* ecotype Oldenburg (Old-1), which still retains the female SI function, showed that *PIP1;4* is up-regulated in compatible pollinations (using wild-type Old-1 pollen), whereas *PIP2;1* and *PIP2;7* are down-regulated in incompatible pollinations (using transgenic self-incompatible Old-1 pollen) (Matsuda et al., 2015). These studies support the hypothesis of pistil **AQP**s potentially regulating pollen hydration in dry stigmas but not in wet stigmas, since the presence of the stigmatic exudate obviates the control of water flow to pollen grains.

**FIGURE 1:**
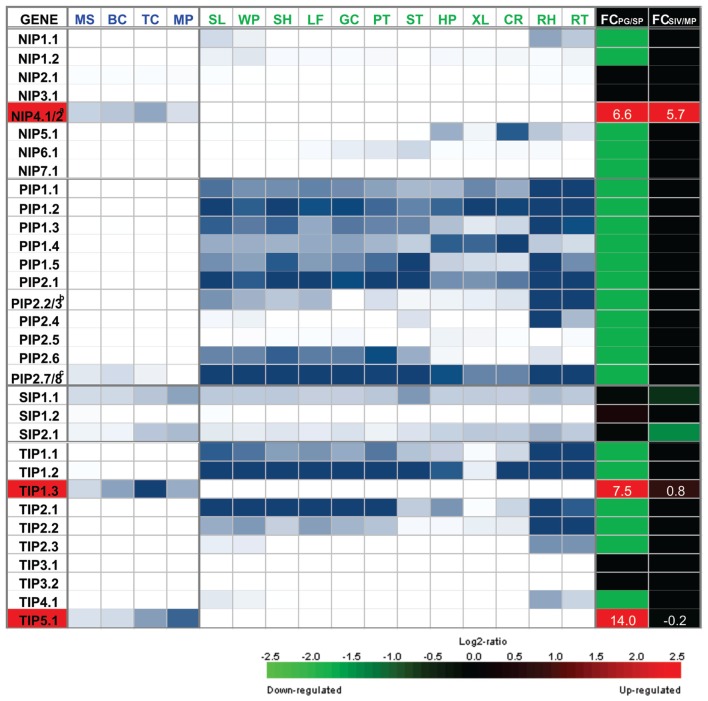
**Heatmap representation of the expression of NIP, PIP, SIP, and TIP genes in gametophytic and sporophytic tissues of *Arabidopsis*.** Microarray data for MS, microspore; BC, bicellular pollen; TC, tricellular pollen; and MP, mature pollen; and for sporophytic organ or tissue, SL, seedlings; WP, whole plants; SH, shoots; LF, leave; GC, guard cell-enriched leaf extracts; PT, petioles; ST, stems; HP, hypocotyls; XL, xylem; CR, cork; RH, root hair zone; RT, roots, was obtained from Bock et al. (2006). Scale: white, 10th percentile, blue, 19th percentile. To identify AQP genes that show specific or preferential expression in pollen, fold change (FC) of the maximum expression from the four pollen grain stages (PG) and the maximum expression from the 12 sporophytic tissues (SP) was calculated as the log2 of the ratio of PG/SP. Microarray data for SIV, pollen tubes grown semi *in vivo*; and MP, mature pollen grains, was obtained from Qin et al. (2009). To identify AQP genes that are differentially expressed upon pollen germination, FC was calculated as the log2 of the ratio of SIV/MP. FC scale: green = -2.5 (down-regulated genes), black = 0, red = 2.5 (up-regulated genes). FC values for pollen specific AQPs NIP4;1/2, TIP1;3, and TIP5;1 are indicated in white. ^a^NIP4;1 and NIP4;2 are promiscuously detected by the same probe set 249584_s_at. Based on RNA-seq data (Loraine et al., 2013) and qRT-PCR assays (Pérez Di Giorgio et al., 2016), pollen grain expression corresponds to NIP4;1; whereas NIP4;2 is only expressed in pollen tubes. ^b^PIP2;2 and PIP2;3 are promiscuously detected by the same probe set 265444_s_at. Based on RNA-seq data (Loraine et al., 2013) none of them are expressed in mature pollen. ^c^PIP2;7 and PIP2;8 are promiscuously detected by the same probe set 266533_s_ during pollen development at BC stage, but based on RNA-seq data (Loraine et al., 2013), they are not expressed in mature pollen.

Analysis performed at four pollen *Arabidopsis* developmental stages confirmed that only TIPs and NIPs, but not *PIPs* are preferentially expressed in mature pollen (Honys and Twell, 2004; Bock et al., 2006) and pollen tubes (Wang et al., 2008; Qin et al., 2009). Genome-wide analysis^1^ of *Arabidopsis* **AQP** genes showed that only four (out of 35 loci) are preferentially expressed in mature pollen and/or pollen tubes: *TIP5;1, TIP1;3, NIP4;1*, and *NIP4;2.* In addition, *AtSIP1;1* and *AtSIP1;2* show both high expression levels in pollen as well as in other sporophytic tissues (Ishikawa et al., 2005), and are therefore not considered to be pollen-specific. *AtPIP2;7/2;8* is expressed during pollen development but has very low levels at maturity, and in turn, shows higher expression levels in sporophytic tissue. *AtTIP1;1* has very low levels of expression in mature pollen, and higher levels in sporophytic tissues. *AtNIP2;1* shows low constitutive levels in pollen and sporophytic tissues, but its expression sharply increase under hypoxic conditions (Choi and Roberts, 2007). It has been demonstrated by *in situ* hybridization and GUS activity assays that *AtNIP7;1* is expressed during pollen development and also in other sporophytic tissues (Li et al., 2011). **Figure 1** shows a heatmap representation of **AQP** expression, which highlights the distinctive repertoire of *Arabidopsis* pollen **AQP**s.

## Seeking their Physiological Role

### TIP1;3 and TIP5;1

*Arabidopsis TIP1;3* and *TIP5;1* are among the most highly expressed genes in mature pollen. *TIP1;3* is expressed in vesicles and vacuoles of vegetative cells while *TIP5;1* is expressed in vacuoles of sperm cells when expressed under its own promoter (Wudick et al., 2014), or in the mitochondria of vegetative cells when heterologously expressed under the control of the LAT52 promoter (Soto et al., 2010). Single *tip1;3* and *tip5;1* mutant plants showed no apparent phenotypic defects in pollen development and no significant reduction in fertility (Soto et al., 2010). However, double *tip1;3 tip5;1* mutant plants showed an abnormal incidence of sterile pods under water- or nitrogen-deficient conditions and heat stress (Wudick et al., 2014). TIP1;3 and TIP5;1 are bi-functional AQPs with intermediate levels of permeability to water and high permeability to urea when expressed in *Xenopus* oocytes (Soto et al., 2008). Interestingly, Soto et al. (2010) showed that TIP5;1 water transport activity is significantly inhibited by an acidic external pH, and therfore proposed that His131, located in extracellular loop C in TIP5;1 and not present in other *Arabidopsis* TIPs, acts as the pH-sensing amino acid. In addition, it was shown that single *tip1;3* and *tip5;1* and double *tip1;3 tip5;1* mutant plants showed shorter pollen tubes only when they were germinated *in vitro* under nitrogen-deficient conditions (Soto et al., 2010). These results suggested that TIP5;1 and TIP1;3 are involved in the nitrogen metabolic pathway during pollen tube growth (Soto et al., 2008, 2010).

### NIP4;1 and NIP4;2

*Arabidopsis NIP4;1* and *NIP4;2* have two distinct features: both are paralog genes found exclusively in the angiosperm lineage and, although they share 84% amino acid identity, they display different expression patterns. Pérez Di Giorgio et al. (2016) found that NIP4;1 is modestly expressed from the unicellular microspore to the mature pollen stage, and functions in pollen development and germination. In turn, NIP4;2 is highly expressed following pollen germination, and functions exclusively during pollen tube growth. NIP4;1 and NIP4;2 are localized in the plasma membrane and internal vesicles of pollen tubes when expressed under their own promoters. In addition, a dynamic cycling between both sub-cellular compartments was observed for NIP4;1. Single *nip4;1* mutant plants showed reduced fertility due to defective pollen development (higher percentages of immature pollen, arrested in uni- or bi-cellular stages, losing viability and collapsing in some cases), pollination and germination; single *nip4;2* mutant plants showed defective pollen tube growth. Double knockdown plants displayed an abnormal incidence of sterile and stunted siliques with fewer seeds as a result of reduced fertilization, owing to defective pollen germination and pollen tube growth. Swelling assays in *Xenopus* oocytes showed that NIP4;1 and NIP4;2 function as water and glycerol channels. In addition, NIP4;1 and NIP4;2 C-termini were found to be phosphorylated by a pollen-specific CPK, modifying their water permeability. Survival assays in yeast indicated that NIP4;1 also transports ammonia, urea, H_2_O_2_ and boric acid. One of the primary functions of boron in plants is to serve in the cross-linking of rhamnogalacturonan-II (RG-II), a component of cell wall pectic polysaccharides, and thus is an essential micronutrient required for plant growth and reproduction (Blevins and Lukaszewski, 1998). Indeed, double knockdown *nip4;1nip4;2* pollen showed shorter pollen tubes, particularly under boron deficient conditions, suggesting that NIP4;1 and NIP4;2 might be involved in boric acid uptake. Likewise, *Arabidopsis* NIP7;1, which is selectively expressed at the microspore stage, was also identified as a boric acid channel, and *nip7;1* mutant plants showed defects in pollen tube growth in the absence of boric acid (Li et al., 2011). Considering that NIP4;1 and NIP4;2are exclusive to angiosperms (Anderberg et al., 2012; Abascal et al., 2014) and that flowering plants are thought to have evolved under selection for a faster reproductive cycle (Williams, 2008, 2012; Abercrombie et al., 2011), NIP4;1 and NIP4;2might contribute to improved pollen germination and pollen tube growth in angiosperms.

### The Working Hypothesis: Relevance of Aquaporins in Plant Fitness

**FIGURE 2:**
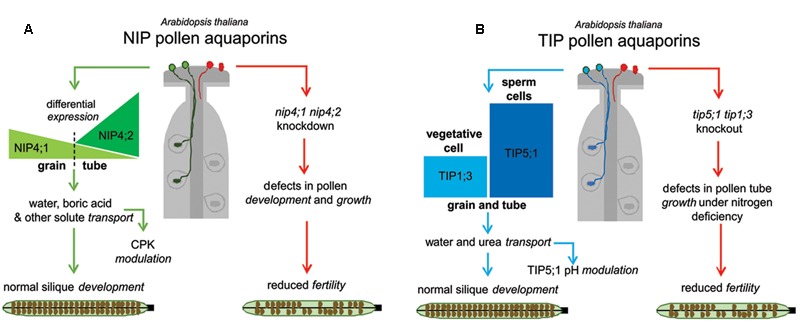
**Global models for the roles of pollen specific aquaporins (AQPs) in reproduction. (A)** In the presence of NIP4;1 and NIP4;2, plant fertility is normal (left, green pathway). NIP4;1 and NIP4;2 expression peaks differ temporally. In the absence of NIPs AQPs, (right, red pathway) plant fitness is reduced (fewer seeds and shorter siliques due to non-fertilized ovules). The *nip4;1nip4;2* knockdown showed defects in pollen development, maturation, germination, and pollen tube growth. **(B)** In the presence of TIP1;3 and TIP5;1 there is normal fertilization (left, blue pathway). TIP1;3 and TIP5;1 expression patterns are also differentially localized in vegetative and sperm cells, respectively. In the absence of both TIPs (right, red pathway), plant fitness is reduced under nitrogen deficiency (in agreement with their substrate specificity) due to defects in pollen tube growth.

Current evidence from the literature shows that specific **AQP**s are part of the machinery of pollen physiology. The fact that *nip4;1* and *nip4;2* mutant phenotypes were evident under normal growth conditions (and not when they are limited) reflects the relevance of these two specific NIPs in reproduction. Although most of the described defects in pollen development, germination and pollen tube elongation are mild at the physiological level, they somehow reveal reduced plant fitness. A model that reflects this hypothesis is proposed in **Figure 2**: when the four **AQP**s are following their normal pattern of expression, plant fertility is not affected. In panel A, NIP4;1 is expressed at relatively low levels in mature pollen, while NIP4;2 expression peaks only after pollen germination. Both NIPs transport water and glycerol (in *Xenopus* oocytes), and in particular NIP4;1 can also transport other solutes such as boric acid, ammonia, H_2_O_2_ and urea in yeast assays. Besides, the water transport capacity of both NIPs is regulated by phosphorylation at Ser267. In the absence of both NIP **AQP**s plant fitness is reduced (less seeds and shorter siliques due to non-fertilized ovules). Single *nip4;1* mutant show defects in pollen development, maturation and germination, while *nip4;2* mutant only in pollen tube growth. In panel B, when TIP1;3 and TIP5;1 are normally expressed, there is also normal fertilization. TIP1;3 is expressed at medium levels in the vegetative cell, while TIP5;1 is the highest gene expressed in sperm cells, where it is the one of the top 200 expressed genes on pollen sperm transcriptomic analysis (Borges et al., 2008). Both TIPs transport water and urea in *Xenopus* oocytes, and water transport of TIP5;1 is regulated by pH. In the absence of both TIPs, plant fitness under nitrogen deficiency is reduced (in accordance with their substrate specificity), due to defects in pollen tube growth.

Another important feature described in certain AQPs is the transport of gas molecules, including carbon dioxide and hydrogen peroxide (Herrera and Garvin, 2011; Bienert and Chaumont, 2014; Kaldenhoff et al., 2014). The physiological relevance of AQP-facilitated gas diffusion includes both the exchange capacity and the signaling process. For instance, hydrogen peroxide (H_2_O_2_), acts as a signaling molecule at lower concentrations, maintaining normal plant growth and development, but it produces toxic effects at higher levels. Specific AQP isoforms are proposed to be critical in the H_2_O_2_ signaling network (Bienert and Chaumont, 2014). Importantly, abiotic stress induces excess accumulation of ROS leading to pollen abortion and programmed cell death of microspores in developing anthers, and consequently resulting in male sterility (Zinta et al., 2016). In this regard, pollen-specific NIP4;1 and other pollen-expressed AQPs, such as TIP1;1, NIP2;1, and NIP7;1 have been shown to transport H_2_O_2_ (revised in Pérez Di Giorgio et al., 2014), and therefore potentially play a role in the redox control of pollen development.

In addition, H_2_O_2_ was shown to mediate redox signaling in pollen-pistil interactions (Sharma and Bhatla, 2013). Considering AtPIP1;4 was found to be up-regulated in stigmas upon compatible pollination (Matsuda et al., 2015) and has a role in H_2_O_2_ transport for signal transduction in immunity pathways (Tian et al., 2016), we could speculate a role of this AQP in ROS signaling during pollination.

## Uncertain Issues that Still Need to be Addressed

The paradox in the analysis of the pollen specific AQPs is that none is an “orthodox” water AQP. Although they differ substantially, TIP5;1, TIP1;3, NIP4;1, and NIP4;2 are solute and water transporters. These findings are consistent with other NIPs and TIPs described in the literature in other organs and/or tissues (Pérez Di Giorgio et al., 2014). In particular, NIPs are considered AQPs with poor water permeability but capacity to transport glycerol, formamide, urea, ammonia or metalloids (Wallace et al., 2002; Danielson and Johanson, 2010; Mitani-Ueno et al., 2011). In agreement with these observations, it was recently proposed that certain NIP channel proteins should be considered metalloido-porins and not AQPs (Pommerrenig et al., 2015).

Even if the analysis of a complete set of pollen AQPs is broadened to include the non-specific SIP1;1 and SIP2;1, it should be emphasized that these two SIPs are preferentially expressed in the endoplasmic reticulum of stems of *Arabidopsis* plants (Maeshima and Ishikawa, 2008).

Thus, pollen tube growth employs a tightly regulated and complex machinery to rapidly coordinate water and solute exchange. Pollen tube growth depends on plasma membrane polarization at the tip involving a strong and rapid membrane recycling to the secretory system (Hepler et al., 2001; Helling et al., 2006). Recently, a highly expressed K^+^ channel (LilKT1) in *Lilium* pollen was found to include a strong endocytic recycling mechanism (Safiarian et al., 2015). The authors propose that this feature could be a way to adjust the number of inward K^+^ channels in the pollen plasma membrane in order to sustain the correct water influx (driven by the raising cytosolic K^+^ concentrations). The fact that the described AQPs move uncharge solutes broadens the discussion of the controlled driving forces that operate in pollen tube growth. Mechano-sensitive channels have also been considered to be potential components in sensing osmotic changes (Zerzour et al., 2009; Kurusu et al., 2013), and these type of channels had already been described in pollen protoplasts (Dutta and Robinson, 2004). A recent study has identified and characterized a pollen-specific membrane tension-gated ion channel, MscS-like 8 (MSL8), which is critical both for pollen survival during the hypoosmotic shock of rehydration and for sustaining full male fertility (Hamilton et al., 2015). There is also new evidence in the literature that certain AQPs are mechanosensitive (human AQP1, described in Ozu et al., 2013). The possibility of analyzing these AQPs as components of an osmosensor system that regulates this machinery might be reasonable but remains speculative (Hill and Shachar-Hill, 2015).

## Author Contributions

GA and JM conceived the idea of this work, JPDG and GA planned and wrote the manuscript, and JM and GS contributed with discussion and critical comments. All authors approved the final version.

## Conflict of Interest Statement

The authors declare that the research was conducted in the absence of any commercial or financial relationships that could be construed as a potential conflict of interest.

## Footnotes

1 https://genevestigator.com/gv/
